# Phase II trial of intralesional therapy with interleukin-2 in soft-tissue melanoma metastases

**DOI:** 10.1038/sj.bjc.6601320

**Published:** 2003-10-28

**Authors:** P Radny, U M Caroli, J Bauer, T Paul, C Schlegel, T K Eigentler, B Weide, M Schwarz, C Garbe

**Affiliations:** 1 Department of Dermatology, Eberhard-Karls-University, Liebermeistersraße 20, Tübingen 72076, Germany

**Keywords:** intralesional therapy, interleukin-2, soft-tissue melanoma metastases, apoptosis

## Abstract

The objective of the present study was to validate the use of intralesional injection of interleukin-2 (IL-2) in patients with skin and soft-tissue melanoma metastases. A total of 24 patients with AJCC stage III or IV melanoma and single or multiple skin and soft-tissue metastases were included. Interleukin-2 injections were administered intralesionally into the total number of cutaneous and soft-tissue metastases accessible from the skin, 2–3 times weekly, over 1–57 weeks. Single doses varied from 0.6 to 6 × 10^6^ IU, depending on lesion size. The clinical response was monitored by sonography and confirmed by histopathology; response evaluation was confined to the intralesionally treated tumours. Complete response (CR) of the treated metastases was achieved in 15 patients (62.5%), the longest remission lasting 38 months to date. In five patients, partial response (PR) was achieved (21%) and in another three patients, progressive disease was observed (one patient not assessable). A total of 245 metastases were treated with CR in 209 (85%), and PR in 21 (6%). The therapy was generally well tolerated; the observed adverse events were mainly of grade 1–2 severity. Immunohistochemical studies showed the tumour cells undergoing apoptosis and revealed a mixed character of the inflammatory infiltrate. The unusual high CR rate in metastatic melanoma of 62.5% and the limited toxicity suggest that treatment of skin and soft-tissue melanoma metastases with intralesional injection of IL-2 may be a safe and effective alternative to conventional therapies. The optimal dosage and duration of this therapy still remain to be defined in larger prospective multicentre trials.

To date, there is no recommended standard therapy for advanced melanoma with skin and soft-tissue metastases. Although a number of therapies, such as surgery, limb perfusion, systemic chemo- or immunochemotherapy, and radiotherapy are currently in use for this situation, management of advanced melanoma with skin and soft-tissue metastases can be a major challenge, particularly in cases of rapidly progressive disease, multiple recurrences, and extensive previous therapy. Surgery is usually restricted to situations with limited disease extension and becomes less indicated with increasing number of recurrences. Systemic chemo- or immunochemotherapies as possible alternatives are also usually of only limited benefit, as skin and soft-tissue metastases often respond poorly and these therapies are commonly associated with severe side effects. Radiotherapy is generally considered as a last resort for advanced melanoma and commonly has only a temporary effect (Pitts and Maloney, 2000; Reeves and Coit, 2000; Walsh *et al*, 2000).

Intralesional therapy modalities generally lack the severe side effects of systemic therapies and, provided sufficient efficacy, can be desirable alternatives to surgery, systemic therapy, or radiotherapy. The therapeutic value of several intralesionally administered cytostatic agents and cytokines, such as fotemustine (Schallreuter *et al*, 1991), bleomycin (Glass *et al*, 1996; Heller *et al*, 1998), cisplatin (Burris *et al*, 1998), interferon-alfa (von Wussow *et al*, 1988), interferon-beta (Fierlbeck *et al*, 1992; Umeda *et al*, 1998; Cornejo *et al*, 2000), and GM-CSF (Vaquerano *et al*, 1999) has been investigated with varying outcome. However, none of these therapies has yet been shown to be a convincing alternative to conventional therapies.

Interleukin-2 (IL-2) was described to be efficient in treating cutaneous and soft-tissue *metastases administered intravenously* (Phan *et al*, 2001) and *administered* intralesionally in a few cases (Gutwald *et al*, 1994a, 1994b). Therefore, we addressed the question of whether intralesional IL-2 is effective in treating soft-tissue metastasis when other therapeutic measures like surgery, limb perfusion, radiotherapy, or chemotherapy had failed to control the disease. Intralesional IL-2 treatment was performed with the aim of achieving a complete regression of all cutaneous and soft-tissue metastases accessible from the skin.

Here, we present the results of a study investigating the feasibility, efficacy, and safety of intralesionally injected IL-2 in 24 melanoma patients with skin and soft-tissue metastases.

## MATERIALS AND METHODS

### Patients

Between November 1998 and April 2002, 24 patients, 14 females and 10 males, aged 19–83 years, with AJCC stage III–IV melanoma, were included in this study. The study protocol was approved by our institutional ethic committee, and patients gave their consent after being informed of the investigational nature of the study. Patients were required to have a life expectancy of 3 months or longer, and no evidence of infection. Patients were not eligible for enrolment into the study if they had grade 3 or 4 cardiac, pulmonary, or CNS disease.

The general characteristics of the patients are summarised in Table 1

**Table 1:** Patient characteristics

Total no. of patients	24
*Gender*	
Male	10 (42%)
Female	14 (58%)
	
*Age (years)*	
Median	59.2
Range	19–83
	
*Stage of disease*	
Metastatic	24 (100%)
	
*American Joint Committee on Cancer*	
Stage III	16 (67%)
Stage IV	8 (33%)
	
*Sites of metastases*	
Cutaneous	2 (8%)
Soft tissue	22 (92%)
Visceral	4 (17%)

. At the time of study inclusion, 16 patients had stage III disease, 15 patients had cutaneous or subcutaneous intransit metastases, and one patient had recurring regional lymph node metastases. In all of them, surgical excisions had been previously performed, and in some of them radiotherapy (two patients) and limb perfusions (three patients) had been additionally applied. Eight patients were included in stage IV disease, two of them with exclusively cutaneous/subcutaneous metastasis, and six with cutaneous metastasis in combination with visceral metastasis. In the 18 patients (16 with stage III disease and two with stage IV disease) with locoregional or exclusively cutaneous metastasis, the total number of soft-tissue metastases accessible from the skin have been included in the intratumoral therapy with IL-2. The six patients with simultaneous visceral metastases had developed their cutaneous/subcutaneous metastases under systemic chemotherapy. They were included because they had the feeling of a stigma by their visible metastases. All patients had received previous therapies for melanoma (summarised in Table 2

**Table 2:** Prior treatment

**Type of treatment**	**No. (%)**
Total no. of patients	24 (100%)
Surgery	24 (100%)
Immunotherapy (IFN-a)	1 (4%)
Chemotherapy (vindesin)	6 (25%)
Chemotharapy (Dacarbazine)	2 (8%)
Immunochemotherapy (Dacarbazine+IFN-a)	6 (25%)
Local radiotherapy	4 (17%)
Limb perfusion (melphalan+dactinomycin)	4 (17%)

): all 24 patients had undergone multiple surgical procedures, six had received systemic chemotherapy with vindesine, another six had received systemic immunochemotherapy with dacarbazine and IFN-alpha (IFN-a), and one had received systemic immunotherapy with IFN-a. Four patients had been treated with localised radiotherapy, and four patients had undergone limb perfusion with dactinomycin and melphalan. None of the patients had received previous IL-2 therapy.

### Study design

Patients were exclusively treated on an outpatient basis at the Department of Dermatology, University of Tuebingen, Germany. For the preparation of stock solution, 18 MIU recombinant human IL-2 (Proleukin®, Chiron, Ratingen, Germany) was dissolved in 6 ml glucose (5%) prepared with albumin (0.2%) solution. The stock solution was injected intralesionally, single doses varied from 0.6–6 MIU (0.2–2.0 ml), depending on the lesion size (see Table 3

**Table 3:** Treatment regimen

**Lesion size maxim. diameter (mm)**	**Single dose MIU**	**Stock solution (ml)**	**Duration of treatment (weeks)**
<5	0.6	0.2	2
>5	1.2	0.4	3
>10	3.0	1.0	4
>20	6.0	2.0	4

). The maximum daily dose was 12 MIU IL-2.

Injections were given intralesionally. Treatment was initially given to the largest metastases until the maximum dose was reached. For deep soft-tissue metastases, sonography was used to guide injections. In the further course, additional smaller metastases were likewise treated. In larger lesions several injections have been applied. Up to 20 small cutaneous lesions have been treated simultaneously. The treatment schedule was 2–3 times weekly for 1–12 weeks. In most of the patients, treatment duration was between 2 and 4 weeks, and the average of injections per lesion was 10 times.

Most patients tolerated the IL-2 injections well, although in single cases the injections were painful. Systemic symptoms have been exclusively treated by metamizole sodium, a common nonsteroidal antipyretic and antiphlogistic. The incidence of adverse events associated with IL-2 treatment was recorded and adverse events were graded according to the WHO criteria.

### Assessment of response and survival probability

The response to treatment was assessed by using the standard criteria of response. The response evaluation has been performed on the basis on an intent-to-treat analysis. Complete response (CR) was defined as disappearance of all clinical evidence of the intralesionally treated tumour. For deep soft-tissue metastases, sonography was used to monitor the clinical response. Additionally, in selected cases biopsies were taken after the completion of therapy for histopathological confirmation of response. Lack of any tumour growth after its clinical regression over a period of at least 6 months was the criterion to classify the result of treatment as CR. Partial response (PR) was defined as a greater than 50% decrease in the sum of product of perpendicular diameters.

Survival curves have been calculated according to the method of Kaplan and Meier separately for the disease stages III and IV taking the diagnosis of the respective disease stage as the start point for the calculation.

### Histopathology and confocal laser scanning microscopy (CLSM)

To confirm complete regression of the tumour by histopathology, biopsies were taken after treatment. Sections of the formalin-fixed and paraffin-embedded tissue were stained with haematoxylin and eosin for routine histopathology. To obtain information on the mechanism of tumour regression, biopsies taken during treatment were studied using immunofluorescence stainings. Vertical paraffin sections (3 *μ*m thick) were pretreated and labelled with anticaspase 3 (rabbit anti-human/mouse-caspase 3, R&D Systems, Wiesbaden, Germany), anti-MART-1/Melan A (monoclonal anti-MelanA, Inno Genex, San Ramon, CA, USA), anti-CD3 (rabbit anti-human-CD3, Linaris, Wertheim, Germany), and anti-CD56 (monoclonal anti-CD56, Novocastra, Dossenheim, Germany). Secondary antibodies were labelled with Cy2, Cy3, and Cy5 (Molecular Probes, Leiden, Netherlands). Nuclei were stained with YOPRO (green) or TOPRO (blue) (Molecular Probes, Leiden, Netherlands). The sections were analysed with a confocal laser scanning microscope (Leica TCS SP, Leica Microsystems, Bensheim, Germany).

## RESULTS

### Response

Of the 24 patients, 23 included in the study were assessable for response. One patient with two treated metastases (patient no. 22 in Table 4

**Table 4:** Treatment details, response, and subsequent course of disease

**Patient**	**Age (years)**	**Sex**	**Stage**	**Number of treated metastases**	**IL-2 dose (MIU)**	**Weeks of treatment**	**Histopathologic response evaluation**	**Local response**	**Subsequent course of systemic disease, systemic therapy**	**Current status**
1	38	F	III	3	281	16	Not done	2 CR, 1 NA	VM (4 M)/T+IFN	DE
2	62	F	III	17	397.5	57	Done	17 CR	Stable	AL, DF
3	69	F	III	5	447	53	Done	5 CR	VM (16 M)/IFN	DE
4	61	M	III	4	61	12	Not done	4 PR	VM (3 M)/T+IFN	DE
5	74	M	III	2	99	8	Not done	2 CR	VM (4 M)/T+IFN	AL, DF
6	59	F	III	3	18	2	Not done	3 CR	Stable	AL, DF
7	83	F	III	1	18	4	Not done	1 CR	VM (6 M)	AL
8	65	M	IV	50	79	8	Done	50 CR	VM (2 M)/T+IFN	DE
9	63	M	III	11	287	18	Done	10 CR, 1 NA	VM (5 M)/IFN	AL
10	58	M	III	1	8	5	Done	1 CR	Stable	AL, DF
11	52	F	IV*	3	115	4	Done	3 CR	Further progression/V	DE
12	30	F	III	3	103	7	Not done	3 CR	Further progression/BOLD	DE
13	35	F	IV*	15	119.2	12	Done	15 PR	Further progression/D+IFN	DE
14	68	M	IV	45	91	4	Done	45 CR	Stable	AL, DF
15	60	F	IV*	7	118	20	Not done	4 NA, 3 PD	Further progression/T	AL
16	66	F	III	5	18	2	Not done	5 CR	Stable	AL, DF
17	64	M	III	1	15.5	2	Done	1 CR	Stable	AL, DF
18	51	F	IV*	4	88	8	Not done	1 PR, 3 PD	Further progression/T+IFN	DE
19	64	F	III	2	175	8	Done	1 CR, 1 PD	VM (7 M)/S+HP	AL, DF
20	78	M	III	20	38.5	12	Done	20 CR	VM (2 M)	AL
21	72	F	III	20	10.5	5	Not done	20 CR	Stable	AL, DF
22	19	M	IV*	2	9	1	Not done	2 NA	Further progression/R	DE
23	59	M	IV*	1	54	3	Not done	1 PR	Further progression/T	AL
24	70	F	III	20	67.5	4	Done	20 CR	Stable	AL, LM

*Stage:* IV^*^=visceral metastases; *IL-2 dose (MIU):* cumulative dose expressed in 10^6^ international units; *Response:* CR=complete response, PR=partial response, NA=not assessable, PD=progression; *Subsequent course of systemic disease:* VM (4 M)=progression to stage IV with visceral metastasis (4 months after commencement of IL-2 therapy), D=dacarbazine, IFN=interferon-alfa, T=temozolomide, V=vindesine, BOLD=polychemotherapy (BOLD regimen), S=surgery, HP=hyperthermic limb perfusion, R=radio therapy; *Current status (July 2000):* Al=alive, DF=disease free, LM=local metastatsis, VM=visceral metastasis, DE=dead.

) was not evaluable for assessing the regression of intralesionally treated lesions, since the injected lesions could not be followed for at least 4 weeks post-treatment due to rapid disease progression and death after 4 weeks.

CR of all intralesionally treated lesions was achieved in 15 patients, the longest remission lasting 38 months to date. In two patients, CR of all except one lesion was achieved, one lesion in each of these patients was classified as not assessable: (1) in patient no. 1 (Table 4) disease progression occurred with visceral metastases, and IL-2 therapy was changed to systemic immunochemotherapy with temozolomide and IFN-a before the response of this single lesions could be assessed. (2) In patient no. 9 (Table 4), a single lesion was surgically excised before completion of IL-2 therapy.

Three patients were classified as PR: in patient no. 4 (Table 4), a definite initial response to IL-2 therapy was seen on sonography. However, when the patient showed disease progression with visceral metastases, IL-2 therapy was changed to systemic immunochemotherapy, but no biopsies of the treated lesions were obtained prior to initiation of systemic therapy to confirm a possible CR. In the second patient classified as partial response (no. 13, Table 4), we had initially classified all 15 lesions as CR. Yet, in one biopsy of a treated lesion, beneath areas of total regression, a small area of vital micrometastasis was seen on histopathology. We therefore reclassified all 15 lesions as PR. The third patient (no. 23 in Table 4) had shown response on ultrasound, unfortunately he developed an abscess at the treated lesion and the region was treated surgically. Therefore, response was classified only as partial.

Altogether, we treated a total of 245 skin and soft-tissue melanoma metastases in 24 patients. We found CR in 209 metastases (85%) and PR in 21 metastases (6%), seven metastases showed progression (3%) and eight metastases (3%) were not assessable. None of the patients with CR developed any local recurrence. We did not observe any systemic response to the local IL-2 treatment, which means we observed no clear regression of any other metastasis simultaneously present, but not intralesionally treated.

### Histopathology and CLSM

**Figure 1:**
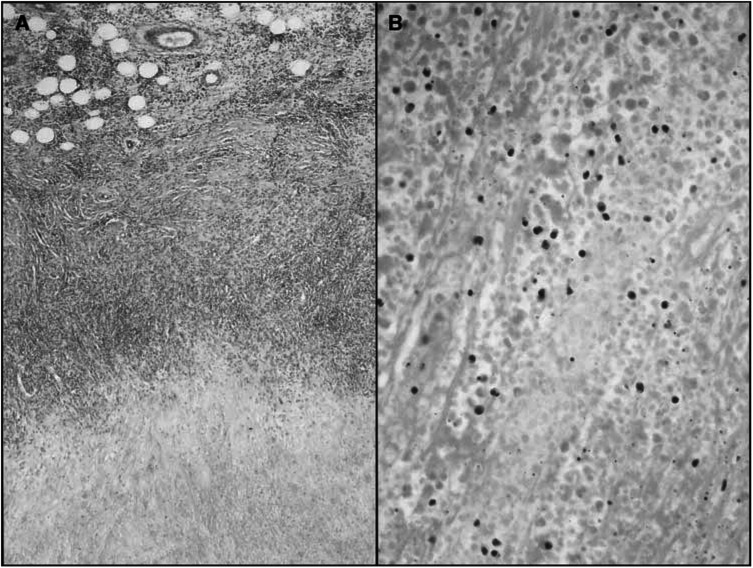
Histology of biopsies taken after the completion of therapy. Complete necrosis of the tumour tissue and a mainly peritumoral lymphocytic infiltrate (**A**) haematoxylin and eosin; original magnification × 40. No viable tumour cells are recognisable; necrotic tumour cells appear as cell shadows. Furthermore, monocytes and macrophages are seen. (**B**) Haematoxylin and eosin; original magnification × 200.

Biopsies were performed in selected cases. The histology of biopsies taken after the end of therapy showed a necrosis of the tumour tissue and an intra- and peritumorous lymphocytic infiltrate. No vital tumour cells could be detected and necrotic tumour cells appeared as cell shadows. Furthermore, monocytes, macrophages, and melanophages were seen (Figure 1A and B

). Only in one single case vital tumour cells forming micrometastasis were found.

**Figure 2:**
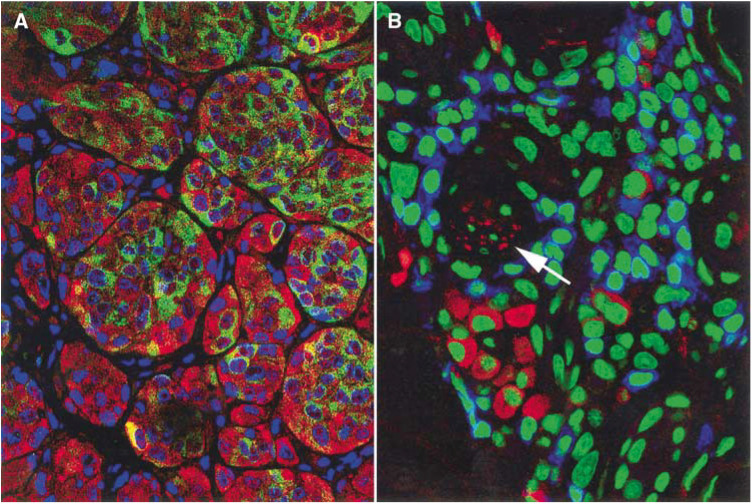
Immunoflourescence staining for Melan A/Mart-1 (green) and caspase-3 (red) detects vital and apoptotic tumour cells. Nuclei stained blue. Images were taken using a CLSM at 630-fold magnification (**A**). Confocal laser scanning microscopy image of the inflammatory infiltrate: CD3-positive T-lymphocytes stained blue, CD56-positive/CD3-negative NK cells stained red, nuclei green, final magnification 630-fold (**B**). Most NK cells are found in small groups near to a capillary (arrow).

Analysis using immunofluorescence techniques and the CLSM revealed a positive staining for caspase 3 in 30–50% of tumour cells already after 1 week of treatment (Figure 2A

). These apoptotic cells increasingly lost their reactivity for anti-Melan A staining. Vital tumour cells were strongly positive for Melan A in most cases. The mononuclear infiltrate was predominantly composed of CD3-postive T cells, and only in some areas CD56-positive, CD3-negative NK cells can be found (Figure 2B). NK cells constitute only a minor proportion of the infiltrate.

### Treatment toxicity

Intralesional IL-2 therapy caused a dose-dependent inflammatory reaction at the site of injection with local swelling and erythema, which completely resolved within days of discontinuation of therapy. On sonography, this IL-2-induced inflammatory reaction could be corroborated as a change in the lesion signal from low-intensity to a slightly higher intensity. Clinically, the inflammatory reaction induced a selective necrosis of the tumour tissue that generally did not affect the surrounding normal tissue. In a few cases (three) with high cumulative IL-2 doses and severe inflammatory reaction, the necrosis exceeded the tumour tissue and resulted in a sterile ulceration of the skin, which healed within weeks of therapy completion.

The incidences of the observed adverse events are given in Table 5

**Table 5:** Adverse events during intralesional IL-2 therapy, graded according to WHO criteria

**Adverse event WHO grade**	**I**	**II**	**III**	**IV**
Local erythema and slight swelling	24 (100%)	0	0	0
Fever	1 (4%)	13 (54%)	0	0
Flu-like symptoms	8 (33)	6 (25%)	0	0
Pain	7 (29)	9 (38%)	0	0
Fatigue	11 (46%)	0	0	0
Nausea/vomiting	8 (33%)	2 (8%)	0	0
Stomach pain	4 (17%)	0	0	0
Diarrhoea	2 (8%)	1 (4%)	0	0
Headache	2 (8%)	0	1 (4%)	0
Muscle cramp	1 (4%)	0	0	0
Tachykardia	1 (4%)	0	0	0

Number of patients and incidence rate.

. The therapy was generally well tolerated. The most common adverse event was local erythema and slight local swelling, which have been observed nearly regularly. Pain due to the injections was also frequent (*n*=16; 67%). The majority of patients described fever (*n*=14; 58%) and flu-like symptoms with chills and night sweats (*n*=14; 58%) that were easily controlled with the use of NSAIDs (metamizole, acetaminophen). Fatigue and nausea or emesis were also common, but usually mild and of short duration. Mild abdominal pain and gastritis-like symptoms, diarrhoea, muscle cramps, and tachycardia were observed in single patients. With the exception of one patient, no grade 3 or 4 adverse events were associated with this therapy. This patient (patient no. 13 in Table 4) experienced severe headache, classified as a grade 3 adverse event, which developed within hours of IL-2 injection on one occasion. The patient had advanced disease with extensive visceral and intra-cerebral metastases that may have exacerbated the headache.

### Subsequent course of disease

**Figure 3:**
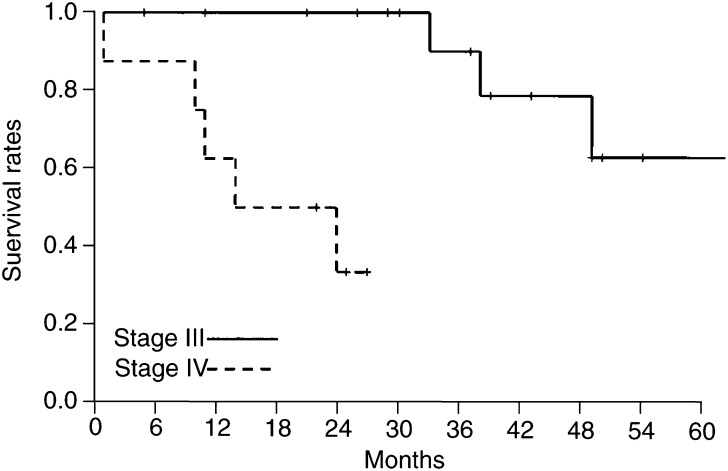
Survival of patients treated with intralesional administered IL-2. Time of diagnosis of stage III respective stage IV disease was taken as the start point for survival curves.

We did not see any recurrences in the treated cutaneous lesions previously responding with CR. However, following intralesional IL-2 therapy, nine of 16 patients in stage III disease progressed to stage IV disease with visceral metastases. The time span between the start of IL-2 therapy and disease progression was 2–16 months. An overview of the subsequent clinical course, treatment, and current status is given in Table 4. Survival curves according to the method of Kaplan and Meier showed that in stage III disease, the 2-year survival rate was 100% and the 5-year survival rate was 63%, and in stage IV disease the 1-year survival rate was 63% and the 2-year survival rate was 33% (Figures 3

and 4

**Figure 4:**
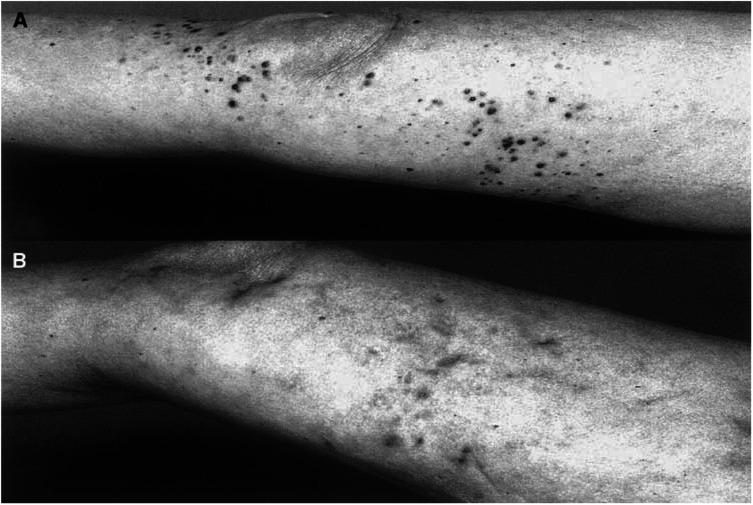
Patient no. 2 in Table 4 with multiple melanoma metastasis at the right leg after two courses of limb perfusion and chemotherapy with vindesine before therapy (**A**) and after 57 weeks treatment, in which each metastasis was individually injected (**B**). In some lesions the response was assessed by histopathology; only residual pigment deposits in the dermis are visible. The patient is under CR to date, currently stable without treatment since 18 months.

).

## DISCUSSION

The results of our pilot study showed that intralesional injection of IL-2 is an effective treatment for soft-tissue melanoma metastases accessible from the skin. The observation of 62.5% CR rate and 83.5% overall response rate is unexpectedly high, and suggests that this new treatment modality may be an interesting alternative to existing therapies. The clinical situation of each patient in this study was challenging at the time of study inclusion, as most patients had rapidly progressive disease or had suffered multiple recurrences. All patients had received extensive previous therapy, including surgery, systemic immuno/chemotherapy, limb perfusion, and radiotherapy, and in the use of surgery had been exhausted.

Intralesional application of antitumorous agents appears as an appealing therapeutic concept, as very high concentrations of the applied agent can be reached within the tumour, which may be essential to attain the desired therapeutic effect. When given systemically, the concentration of IL-2 within the tumour is several magnitudes lower than under intralesional therapy. However, IL-2 administered intravenously was observed in a subgroup of 28 patients with subcutaneous and cutaneous melanoma metastases respectable 53% responder (Phan *et al*, 2001). The resounding effect of intralesional IL-2, which we found in this study was not observed with systemic IL-2 therapy, and is therefore most likely a result of the high intratumoral IL-2 concentration. Furthermore, with intralesional therapy the cumulative IL-2 doses are generally lower than with systemic therapy and as a result, the severe side effects of systemic IL-2 therapy (Keilholz *et al*, 1998; Legha *et al*, 1998; Eton *et al*, 2000; Phan *et al*, 2001) were not observed in our study.

A number of reports of treatment of melanoma metastases with intralesional application of agents with direct or mediated antitumoral effect have been published. Several cytostatic agents, including fotemustine (Schallreuter *et al*, 1991), bleomycin (Glass *et al*, 1996; Heller *et al*, 1998), and cisplatin (Burris *et al*, 1998) have been used intralesionally, with varying results. As immune mechanisms have been shown to play a significant role in the pathogenesis and clinical course of melanoma (reviewed in Curiel-Lewandrowski and Demierre, 2000), a number of reports have addressed the treatment of melanoma metastases with intralesionally injected immunemodulatory cytokines, including interferon-alpha (von Wussow *et al*, 1988), interferon-beta (Fierlbeck *et al*, 1992; Umeda *et al*, 1998; Cornejo *et al*, 2000), and GM-CSF (Vaquerano *et al*, 1999). However, with none of these cytokines satisfactory results were achieved. The successful use of intralesional IL-2 in the treatment of malignant tumours, including melanoma metastases (Gutwald *et al*, 1994a,1994b), squamous cell carcinoma (Whiteside *et al*, 1993), metastatic eccrine poroma (Dummer *et al*, 1992), malignant haemangioendothelioma (Inadomi *et al*, 1992), carcinoma erysipeloides (Hamamoto *et al*, 2001), and in newer times with PEG-IL-2 on basal cell carcinoma has only been published in single case reports (Kaplan and Moy, 2000). Our study is the first to evaluate the feasibility, efficacy, and safety of intralesional IL-2 in a phase II trial in 24 patients.

The most likely mechanism of tumour regression is the creation of lymphokine-activated killer cells (LAK cells) by IL-2 and subsequent destruction of the tumour by the ability of LAK cells to lyse tumour cells or to induce apoptosis directly. LAK cells are derived from either NK cells or from CD8-positive T-cells (Abbas *et al*, 1994). They have been shown to play a critical role in the antitumoral immune response *in vitro* and *in vivo* (Ferlazzo *et al*, 1997; Chikamatsu *et al*, 1999; Hajkova *et al*, 1999; de Gast *et al*, 2000). Indeed, on the histology of biopsies taken from IL-2-treated metastases, we found a dense intra- and peritumoral lymphocytic infiltrate surrounding and infiltrating the areas of necrotic tumour cells. Using CLSM, we could show that tumour cells undergo apoptosis and that the mononuclear infiltrate mainly consists of T cells and to a minor extent of NK cells. So in the setting of intratumoral IL-2 treatment, LAK cells are supposed to be activated T cells inducing apoptosis in tumour cells. Further studies on the mechanism of tumour regression induced by local treatment with IL-2 are needed.

It is difficult to judge whether the intralesional IL-2 treatment is suitable to contribute to prolongation of survival, as survival was not an end point of the study protocol. The survival curves calculated for this patient collective seem to be favourable, but do not allow definitive conclusions. Most importantly, they do not hint at any kind of life-shortening effects of the intratumoral IL-2 therapy.

In conclusion, our study has documented the feasibility of intralesional IL-2 therapy in melanoma patients with skin and soft-tissue metastases. The overall response rate of 62.5% and its limited toxicity suggest that this new therapy may be a suitable alternative to systemic dacarbazine treatment in this particular subset of melanoma patients. The optimal dosage and duration of this therapy still remain to be defined in larger prospective multicentre trials. An interesting question is whether intralesional IL-2 therapy may also be effective in the treatment of metastases of other solid tumours.
